# The Background of Mitochondrial DNA Haplogroup J Increases the Sensitivity of Leber's Hereditary Optic Neuropathy Cells to 2,5-Hexanedione Toxicity

**DOI:** 10.1371/journal.pone.0007922

**Published:** 2009-11-19

**Authors:** Anna Ghelli, Anna Maria Porcelli, Claudia Zanna, Sara Vidoni, Stefano Mattioli, Anna Barbieri, Luisa Iommarini, Maria Pala, Alessandro Achilli, Antonio Torroni, Michela Rugolo, Valerio Carelli

**Affiliations:** 1 Dipartimento di Biologia Evoluzionistica Sperimentale, Università di Bologna, Bologna, Italy; 2 Dipartimento di Medicina Interna, dell'Invecchiamento e Malattie Nefrologiche, Sezione di Medicina del Lavoro, Università di Bologna, Bologna, Italy; 3 Dipartimento di Scienze Neurologiche, Università di Bologna, Bologna, Italy; 4 Dipartimento di Genetica e Microbiologia, Università di Pavia, Pavia, Italy; 5 Dipartimento di Biologia Cellulare e Ambientale, Università di Perugia, Perugia, Italy; Auburn University, United States of America

## Abstract

Leber's hereditary optic neuropathy (LHON) is a maternally inherited blinding disease due to mitochondrial DNA (mtDNA) point mutations in complex I subunit genes, whose incomplete penetrance has been attributed to both genetic and environmental factors. Indeed, the mtDNA background defined as haplogroup J is known to increase the penetrance of the 11778/ND4 and 14484/ND6 mutations. Recently it was also documented that the professional exposure to n-hexane might act as an exogenous trigger for LHON. Therefore, we here investigate the effect of the n-hexane neurotoxic metabolite 2,5-hexanedione (2,5-HD) on cell viability and mitochondrial function of different cell models (cybrids and fibroblasts) carrying the LHON mutations on different mtDNA haplogroups. The viability of control and LHON cybrids and fibroblasts, whose mtDNAs were completely sequenced, was assessed using the MTT assay. Mitochondrial ATP synthesis rate driven by complex I substrates was determined with the luciferine/luciferase method. Incubation with 2,5-HD caused the maximal loss of viability in control and LHON cells. The toxic effect of this compound was similar in control cells irrespective of the mtDNA background. On the contrary, sensitivity to 2,5-HD induced cell death was greatly increased in LHON cells carrying the 11778/ND4 or the 14484/ND6 mutation on haplogroup J, whereas the 11778/ND4 mutation in association with haplogroups U and H significantly improved cell survival. The 11778/ND4 mutation on haplogroup U was also more resistant to inhibition of complex I dependent ATP synthesis by 2,5-HD. In conclusion, this study shows that mtDNA haplogroups modulate the response of LHON cells to 2,5-HD. In particular, haplogroup J makes cells more sensitive to its toxic effect. This is the first evidence that an mtDNA background plays a role by interacting with an environmental factor and that 2,5-HD may be a risk element for visual loss in LHON. This proof of principle has broad implications for other neurodegenerative disorders such as Parkinson's disease.

## Introduction

Leber's hereditary optic neuropathy (LHON) is a maternally inherited blinding disease due to three most frequent mitochondrial DNA (mtDNA) point mutations (11778/ND4, 3460/ND1 and 14484/ND6), which affect complex I subunit genes [1]. In most LHON pedigrees the causative mtDNA mutation is homoplasmic (100% mtDNA copies are mutant) in all maternally related individuals, but only a subset of them develops the optic neuropathy, usually estimated as about 50% of the males and 10% of females [2]. The incomplete penetrance is assumed to depend on further genetic and/or environmental factors, which may play a role in triggering the visual loss and optic atrophy [3].

Among the genetic factors, there is now a solidly established evidence that a Western Eurasian mtDNA background, known as haplogroup J, plays a modifying role increasing the pathogenic potential and hence the penetrance of the 11778/ND4 and 14484/ND6 LHON mutations [4], [5]. The modifying effect of haplogroup J is thought to be due to specific arrays of complex I and III non-synonymous polymorphisms characterizing sub-haplogroup J1 for the 14484/ND6 mutation and sublineages J1c and J2b for the 11778/ND4 mutation [4]. The functional alteration underlying this modifying effect has not been clearly elucidated, but based on accumulation of non-synonymous variants in complexes I and III, instability of supercomplexes has been originally hypothesized [4], [6]. However, recent studies indicated an alternative mechanism to explain the association of haplogroup J variants and LHON penetrance [7]. Furthermore, there is experimental evidence that different mtDNA haplogroups may maintain similar efficiencies of the respiratory function [8], thanks to different settings of reactive oxygen species (ROS) production and control of mitochondrial biogenesis [9]. Transcription and replication of mtDNA have also been proposed to be influenced by specific variants in the D-loop region, again involving haplogroup J [10]. The mtDNA background effect seems to be only one of the possible modifying genetic factors, in fact recent linkage analysis data suggest that one or more genes on chromosome X may also affect LHON penetrance, possibly explaining the male prevalence as well [11], [12].

Environmental factors may also modulate LHON penetrance. Until recently, most of the data presented in literature are still scattered anecdotal reports. Tobacco smoking and alcohol consumption have been proposed as possible triggers for LHON [13]–[15], particularly because their combination has been associated with a form of optic neuropathy, i.e. tobacco-alcohol amblyopia [16], which in some cases was shown to be misdiagnosed LHON [17]. However, a recent epidemiologic study of a large cohort of LHON families reached a well-supported conclusion that tobacco smoking is indeed a triggering factor [18]. In a similar manner, exposures to agricultural pesticides, smoke, toxic vapours from industrial solvents have been variously mentioned as possible environmental triggers for LHON [15], [19]. Recently, we also documented one case, a subject carrying the 11778/ND4 mutation [20], for whom the combined exposure to n-hexane and other solvents apparently acted as the trigger for the optic neuropathy.

The association between occupational exposure to solvents and neurological complications involving the retina and the optic nerve is well established [21], [22]. In a cross-sectional study on 15 workers exposed to n-hexane, 11 showed macular changes and 1 had central retinopathy [23]. Glue sniffers may also suffer optic neuropathy and/or hearing loss [24]–[26]. Exposure to (or sniffing of) toluene has been implicated in acute disturbances of color vision and retinal and optic nerve degeneration [27], [28]. Moreover, animal models of n-hexane exposure identified 2,5-hexanedione (2,5-HD) as a neurotoxic n-hexane metabolite [29], inducing deterioration in visual function [30]. Studies on n-hexane toxicity showed uncoupling of mitochondrial respiration [31]. Furthermore, co-exposure and possible interactions between solvents and/or their metabolites may synergize the potential neurotoxicity accumulating in different tissues [32], [33].

In the present study we have investigated the toxic effect of 2,5-HD in cybrids and fibroblasts bearing LHON mutations and evaluated the involvement of complex I. We also assessed whether toluene further increases the 2,5-HD toxicity. Our results were evaluated in relation to the sequence variation and the haplogroup affiliation of the mtDNA on which the LHON pathogenic mutation was present.

## Results

### Genetic Characterization of the Cells

We employed the well established cell model for mtDNA functional studies called cybrids (transmitochondrial cytoplasmic hybrids), which has the advantage to maintain a constant nuclear background and use different mtDNAs [34]. However, nuclear genome variation may also have specific interactions with the mtDNA, thus primary cell lines derived from LHON patients, such as fibroblasts, containing both the original nuclear and mitochondrial genomes were also used.

Control and LHON cybrids are the same already characterized in previous investigations, with all LHON cybrids being homoplasmic mutant for one of the three primary mutations [7], [8], [35]–[38]. For some of the cybrid clones the complete mtDNA sequence was already available [7], but for the others has been determined in the current study (Table 1).

**Table 1:** Haplogroup affiliation and non-synonymous nucleotide changes of mtDNAs from LHON and control cybrid and fibroblast cell lines used in this study.

Cell lines	GenBank ID	LHON mutation	Haplogroup	Non-synonymous polymorphisms relative to CRS^a^	Amino acid change
***Cybrids***					
HGA	GQ304740	Control	J1c	T4216C (*ND1*)	Tyr>His
				A10398G (*ND3*)	Thr>Ala
				A13681G (*ND5*)	Thr>Ala
				G13708A (*ND5*)	Ala>Thr
				C14766T (*CYTB*)	Thr>Ile
				T14798C (*CYTB*)	Phe>Leu
				C15452A (*CYTB*)	Leu>Ile
HPC	EU915472 [7]	Control	H5	A7245G (*COI*)	Thr>Ala
HPS	GQ304741	Control	T2	T4216C (*ND1*)	Tyr>His
				A4917G (*ND2*)	Asn>Asp
				C10750T (*ND4L*)	Asn>Ser
				C14766T (*CYTB*)	Ile>Thr
				C15452A (*CYTB*)	Leu>Ile
HPE	EU915476 [7]	G11778A (*ND4*)	J1c	T4216C (*ND1*)	Tyr>His
				A10398G (*ND3*)	Thr>Ala
				T12083G (*ND4*)	Ser>Ala
				G13708A (*ND5*)	Ala>Thr
				C14766T (*CYTB*)	Thr>Ile
				T14798C (*CYTB*)	Phe>Leu
				C15452A (*CYTB*)	Leu>Ile
HFF	EU915477 [7]	G11778A (*ND4*)	U5a1	G9477A (*COIII*)	Val>Ile
				A9667G (*COIII*)	Asn>Ser
				C14766T (*CYTB*)	Thr>Ile
				A14793G (*CYTB*)	His>Arg
RJ206	GQ304742	G3460A (*ND1*)	T1a	T4216C (*ND1*)	Tyr>His
				A4917G (*ND2*)	Asn>Asp
				C14766T (*CYTB*)	Thr>Ile
				C15452A (*CYTB*)	Leu>Ile
HMM12	EU915475 [7]	G3460A (*ND1*)	H12	A14552G (*ND6*)	Val>Ala
HL180	EU915479 [7]	T14484C (*ND6*)	J1c	T4216C (*ND1*)	Tyr>His
				T7042C (*COI*)	Val>Ala
				A10398G (*ND3*)	Thr>Ala
				G13708A (*ND5*)	Ala>Thr
				G14279A (*ND6*)	Ser>Leu
				C14766T (*CYTB*)	Thr>Ile
				T14798C (*CYTB*)	Phe>Leu
				C15452A (*CYTB*)	Leu>Ile
HBA2	EU915478 [7]	T14484C (*ND6*)	J1b	T4216C (*ND1*)	Tyr>His
				G5460A (*ND2*)	Ala>Thr
				G8557A (*ATP6*)	Ala>Thr
				A10398G (*ND3*)	Thr>Ala
				G13708A/(ND5)	Ala>Thr
				T13879C/(ND5)	Ser>Pro
				C14766T (*CYTB*)	Thr>Ile
				C15452A (*CYTB*)	Leu>Ile
**Fibroblasts**					
C88/F03W	GQ304743	Control	J1c	T4216C (*ND1*)	Tyr>His
				A10398G (*ND3*)	Thr>Ala
				G13708A (*ND5*)	Ala>Thr
				C13934T (*ND5*)	Thr>Met
				C14766T (*CYTB*)	Thr>Ile
				T14798C (*CYTB*)	Phe>Leu
				C15452A (*CYTB*)	Leu>Ile
KM/F08W	GQ304744	Control	T2b	T4216C (*ND1*)	Tyr>His
				A4917G (*ND2*)	Asn>Asp
				G9948A (*COIII*)	Val>Ile
				T14766T (*CYTB*)	Thr>Ile
				C15452A (*CYTB*)	Leu>Ile
VS/F07W	GQ304745	Control	H*	none	
FJ/F08L	GQ304746	G11778A (*ND4*)	J1c	T4216C (*ND1*)	Tyr>His
				G9145A (*ATP6*)	Ala>Thr
				A10398G (*ND3*)	Thr>Ala
				G13708A (*ND5*)	Ala>Thr
				A13933G (*ND5*)	Thr>Ala
				C14766T (*CYTB*)	Thr>Ile
				T14798C (*CYTB*)	Phe>Leu
				C15452A (*CYTB*)	Leu>Ile
RA/F07L	GQ304747	G11778A (*ND4*)	H*	A7904G (*COII*)	Thr>Ala
				G14249A (*ND6*)	Ala>Val
CC/F09L	EF060364 [44]	G3460A (*ND1*)	U4a	C14766T (*CYTB*)	Thr>Ile
				T15693C (*CYTB*)	Met>Thr
				G15773A (*CYTB*)	Val>Met
WR/F30L	GQ304748	T14484C (*ND6*)	V	none	
HL/F29L b	EU915479 [7]	T14484C (*ND6*)	J1c	T4216C (*ND1*)	Tyr>His
				T7042C (*COI*)	Val>Ala
				A10398G (*ND3*)	Thr>Ala
				G13708A (*ND5*)	Ala>Thr
				G14279A (*ND6*)	Ser>Leu
				C14766T (*CYTB*)	Thr>Ile
				T14798C (*CYTB*)	Phe>Leu
				C15452A (*CYTB*)	Leu>Ile

a rCRS refers to the revised Cambridge reference sequence [58]. In addition, all mtDNAs differed from rCRS, which belongs to haplogroup H2a, for A8860G (*ATP6*) and A15326G (*CYTB*).

b This fibroblast cell line is from the same patient and harbours the same mtDNA as cybrid HL180.

The LHON fibroblasts were also homoplasmic mutant for one of the three primary mutations. The complete mtDNA sequence was also determined for both control and LHON fibroblasts (Table 1). For each LHON and control cell line (cybrids and fibroblasts), the haplogroup affiliation and the full list of non-synonymous mtDNA mutations are listed in Table 1.

### Viability Experiments with 2,5-HD in Cybrids

**Figure 1:**
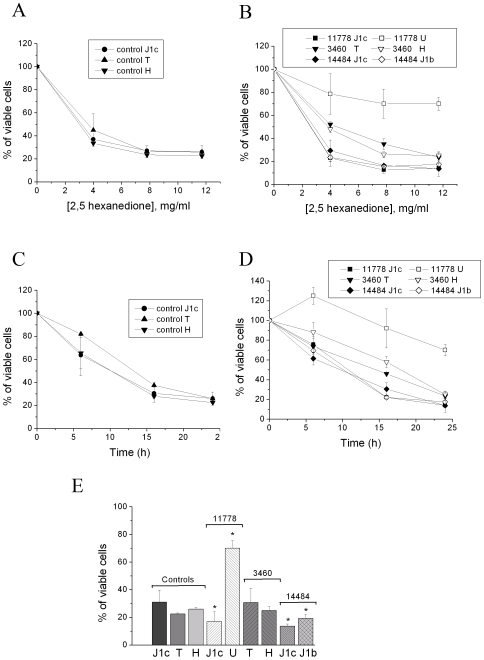
Effect of 2,5-HD on viability of control and LHON cybrids with mtDNAs belonging to different haplogroups. Dose-responses of viability of control (A) and LHON (B) cybrids, with the respective mtDNA haplogroups, incubated for 24 hours in DMEM containing the indicated amounts of 2,5-HD. Time-courses of viability of control (C) and LHON (D) cybrids incubated with 12 mg/ml 2,5-HD. (E) Statistical analysis of the same data obtained in control and LHON cybrids, incubated for 24 hours with 12 mg/ml 2,5-HD. Cell viability was determined and statistically analyzed as described in the Methods section. Data are means±SD of at least 3 determinations. *denotes significantly different values (p<0.05) determined by One Way ANOVA followed by the Holm-Sidak method.

The 2,5-HD toxicity was analyzed by determining the dose-response relationship in control (Figure 1A) and LHON cybrids (Figure 1B). The loss of viability induced by 24 hours incubation with different concentrations of 2,5-HD was independent of the mtDNA background in control cybrids (Figure 1A). On the contrary, LHON cybrids showed a mtDNA haplogroup dependent behaviour (Figure 1B). The LHON cybrid clone carrying the 11778/ND4 mutation on haplogroup U was the most resistant to the toxic effect of 2,5-HD (70% of cells still viable with 12 mg/ml), whereas the LHON cybrid clones on haplogroup J1 were the most sensitive (only 15% of cells viable). In particular, the LHON cybrid clones carrying the 14484/ND6 mutation on haplogroup J1 (J1c and J1b) were as sensitive to 2,5-HD as the one carrying the 11778/ND4 mutation on a similar background (J1c). LHON cybrid clones bearing the 3460/ND1 mutation on haplogroups T and H were similar to controls. The time-dependent changes of viability in control and LHON cybrids, preincubated with 12 mg/ml of 2,5-HD reported in figures 1C and 1D, showed a similar behaviour. Figure 1E summarizes the percentages of viable cells after 24 hours incubation with 12 mg/ml of 2,5-HD, showing that the 11778/ND4 and 14484/ND6 LHON mutations in combination with haplogroup J1 were significantly more sensitive to the toxic effect relative to controls. On the contrary, the cybrid clone harbouring the 11778/ND4 mutation on haplogroup U was significantly associated with a marked resistance to 2,5-HD toxicity.

### ATP Synthesis Rate in Cybrids Treated with 2,5-HD

We preliminarily attempted to measure the complex I activity in cybrids to test if the toxic effect of 2,5-HD was mainly mediated through this pathway, which is impaired by the LHON mutations. However, we verified a low reproducibility of this assay, in particular of the titration of 2,5-HD on complex I activity. Alternatively, we decided to assess the ATP synthesis driven by substrates of both complex I (pyruvate and malate) and complex II (succinate) to investigate if and how the 2,5-HD toxic effect was mediated by inhibition of the mitochondrial respiratory chain. This analysis was restricted to the LHON cybrid clones that were significantly different from controls in the viability assay as reported in figure 1E.

**Figure 2:**
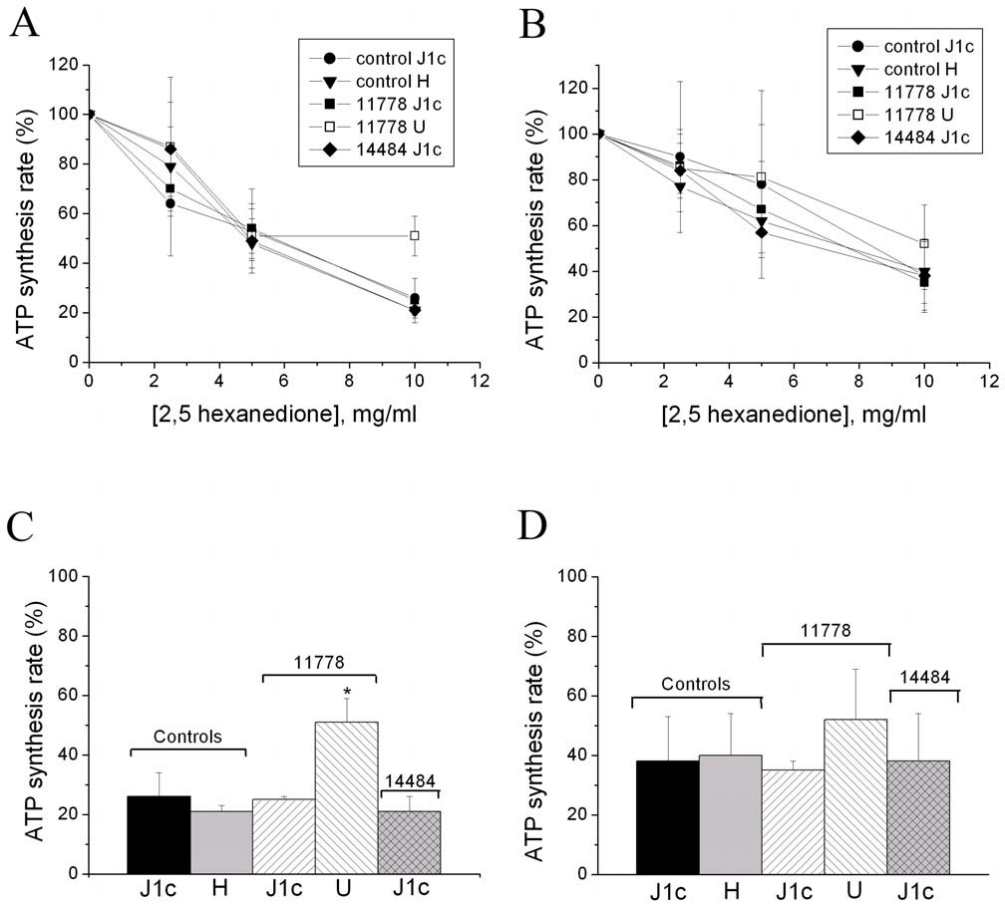
Effect of 2,5-HD on ATP synthesis of control and LHON cybrids with mtDNAs belonging to different haplogroups. Dose-responses of ATP synthesis rate driven by complex I substrates (A) and complex II substrate (B) in digitonin-permeabilized cybrids after addition of the indicated amounts of 2,5-HD. Data are expressed as percentage of the values obtained in untreated samples. Statistical analysis of the same data of ATP synthesis rate obtained in control and LHON cybrids incubated with 10 mg/ml 2,5-HD in the presence of complex I substrates (C) or complex II substrate (D). Data are means±SD of at least 3 determinations. *denotes significantly different values (p<0.05), determined by One Way ANOVA followed by the Holm-Sidak method.

Figures 2A and B show the 2,5-HD dose–response of ATP synthesis driven by complex I and II substrates after addition of 2,5-HD to digitonized cybrids. 2,5-HD inhibited the rate of ATP synthesis mediated by both the respiratory complexes, although its effect was more relevant for complex I. In particular Figure 2A illustrates that the inhibitory effect of 2,5-HD was maximal at the concentration of 10 mg/ml for all cybrid clones, except for that bearing the 11778/ND4 mutation on haplogroup U. The results obtained at 10 mg/ml 2,5-HD are summarized in figures 2C and D. The statistical analysis reveals that the ATP synthesis through complex I in cybrids with the 11778/ND4 mutation on haplogroup U only was significantly less sensitive to 2,5-HD (Figure 2C). The same analysis carried out for complex II substrate showed a similar inhibition of ATP synthesis in all cybrid clones (Figure 2D), indicating that complex I might be seems specifically affected by 2,5-HD.

### Viability Experiments with 2,5-HD in Fibroblasts

The cybrid cell model allows studying the effect of mtDNA mutations in the context of the same nuclear background [34]. However, it has been reported that the cybridization process causes a substantial cellular stress and that genetic instability could produce variability in gene expression [39]. Furthermore, specific variability in the interaction of nuclear and mitochondrial genomes has been also reported and the coevolution of the two genomes debated [40], [41]. We therefore decided to test the effect of 2,5-HD also in a set of control and LHON fibroblasts stratifying again the results according to the haplogroup affiliation of their mtDNAs.

**Figure 3:**
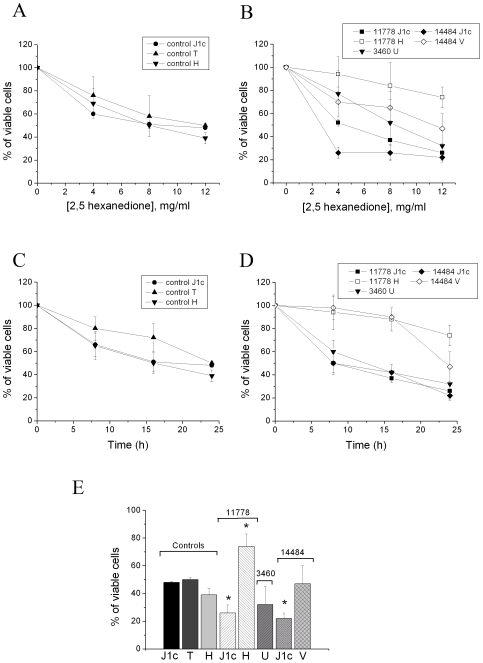
Effect of 2,5-HD on viability of control and LHON fibroblasts with mtDNAs belonging to different haplogroups. Dose-responses of viability of control (A) and LHON (B) fibroblasts, with the respective mtDNA haplogroups, incubated for 24 hours in DMEM containing the indicated amounts of 2,5-HD. Time-courses of viability of control (C) and LHON (D) fibroblasts incubated with 12 mg/ml 2,5-HD. (E) Statistical analysis of the same data obtained in control and LHON fibroblasts, incubated for 24 hours with 12 mg/ml 2,5-HD. Cell viability was determined and statistically analyzed as described in the Methods section. Data are means±SD of 3 determinations. *denotes significantly different values (p<0.05), determined by One Way ANOVA followed by the Holm-Sidak method.

Figure 3A shows that 24 hours incubation with different concentrations of 2,5-HD reduced the viability of control fibroblasts independently from the mtDNA background. On the contrary, LHON fibroblasts carrying the 11778/ND4 mutation on haplogroup H were the most resistant to the toxic effect of 2,5-HD (at 12 mg/ml approximately 70% of cells still viable), whereas the LHON fibroblasts on haplogroup J1 were the most sensitive (Figure 3B). The behaviour of fibroblasts with the 14484/ND6 mutation bearing haplogroup V and with the 3460/ND1 mutation on haplogroup U was similar to controls. The time-dependent changes of viability in control and LHON fibroblasts treated with 12 mg/ml of 2,5-HD (figure 3C and 3D) reveals that, consistently with the other experiments, the 11778/ND4 and 14484/ND6 mutations associated with haplogroup J1 were the most sensitive. Furthermore, the 11778/ND4 mutation with haplogroup H and the 14484/ND6 with haplogroup V were similarly resistant up to 16 hours incubation, whereas at 24 hours the viability of this latter cell line decreased to values similar to controls. Figure 3E summarizes the results obtained after 24 hours of incubation with 12 mg/ml of 2,5-HD, showing that a similar decrease in viable cells (about 50%) was observed in control fibroblasts with haplogroups J1, T, and H. Conversely, LHON fibroblasts carrying the 11778/ND4 or 14484/ND6 mutations in association with haplogroup J1 were again significantly more sensitive to the toxic effect of 2,5-HD. Interestingly, LHON fibroblasts with the 11778/ND4 mutation on a haplogroup H background were markedly more resistant to the toxicity of this compound, whereas no differences were observed with the other mutation/haplogroup combinations relative to controls.

### Viability Experiments with Combined 2,5-HD and Toluene

**Figure 4:**
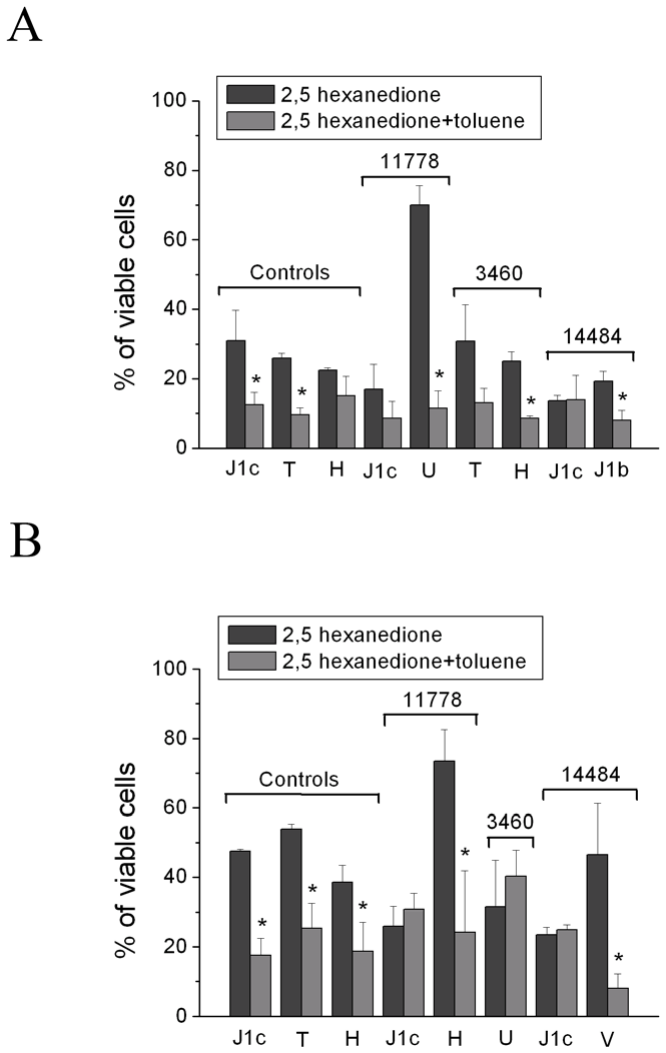
Viability of control and LHON cells after incubation with 2,5-HD and toluene. Cybrids (A) and fibroblasts (B) were incubated for 24 hours in DMEM containing 12 mg/ml 2,5-HD alone or in the presence of 5 mg/ml toluene. Cell viability was determined as described in figure 1. Data are means±SD of 5 determinations. *denotes significantly different values (p<0.05) between cells treated with 2,5-HD alone or in the presence of toluene, using the Student's *t* test.

We further evaluated whether cells were differently affected by the simultaneous incubation with 2,5-HD and toluene, based on our previous report on a LHON patient who developed the disease after exposure to a combination of different organic solvents [20]. To this end a preliminary experiment was carried out by the head-space/SPME/GC-MS method to determine the greatest concentration of toluene soluble in DMEM, with and without 2,5-HD. We established that the maximum concentration of toluene soluble in DMEM was 0.5 mg/ml, which increased to 0.6 mg/ml in the presence of 2,5-HD (data not shown). Cell viability was than evaluated after 24 hours incubation with both 2,5-HD (12 mg/ml) and toluene (0.6 mg/ml). Figure 4A shows that the final mixture caused a marked decrease of viability in all cybrids, except in those bearing the 11778/ND4 or 14484/ND6 mutations with haplogroup J1c. For the control cybrid clone with haplogroup H and the LHON 3460/ND1 cybrid clone on haplogroup T, only a non-significant tendency was observed for the additive effect of toluene. A similar additive effect was also observed in fibroblasts (Figure 4B), except for the 3460/ND1 mutation on haplogroup U and again the 11778/ND4 or 14484/ND6 mutations with haplogroup J1c. It is worth noting that exposure to toluene alone had no effect on cell viability (data not shown).

## Discussion

The current study shows that mtDNA genetic variation, defined in terms of haplogroups, and environmental factors may interact becoming relevant to the pathogenesis of a human disease. The proof of principle of this interaction has broad implications on disease predisposition. In the specific case we applied the paradigm of a mitochondrial disorder dependent on mtDNA point mutations, for which there was an established evidence that certain mtDNA backgrounds play a role to modulate penetrance [4], [5]. Our results have a two-fold relevance for LHON patients. First, 2,5-HD has a detrimental effect on cell viability. Second, the sensitivity to this toxicant of cells carrying the 11778/ND4 and 14484/ND6 LHON pathogenic mutations is modified by the mtDNA background, being haplogroup J1 the most sensitive. Haplogroup J is also the same mtDNA background previously associated with an increased penetrance for these LHON mutations [4], [5].

By studying cells under the assumption of a constant nuclear background, in which different mtDNAs were introduced, we observed that haplogroup J1 was the most sensitive to the toxicant, when the LHON pathogenic mutations 11778/ND4 or 14484/ND6 were present. Only the combination of these LHON pathogenic mutations with haplogroup J1 background increased the sensitivity to 2,5-HD, as shown by the direct comparison with control cybrids carrying haplogroup J1 mtDNA without the LHON mutations. Moreover, we observed that the single cybrid clone carrying the 11778/ND4 mutation on haplogroup U behaved as the most resistant to the toxicant effects. Unfortunately, we could not mirror our results on haplogroup J1 for the LHON cell line carrying haplogroup U, not having available a control cybrid cell line with a haplogroup U mtDNA for direct comparison.

By direct assessment of the ATP synthesis rate, we also demonstrated that the neurotoxin 2,5-HD inhibited the oxidative phosphorylation. Previous studies documented a direct effect of the n-hexane metabolite 2,5-HD at the mitochondrial level. In fact, inhibition of state 3 respiration was reported after 2,5-HD addition to isolated brain mitochondria and also after chronic treatment of rats [42]. Furthermore, 2,5-HD was shown to induce apoptotic death in spermatogenic cells through a mitochondrial pathway, involving loss of mitochondrial membrane potential [43]. We report here that the 2,5-HD effect was more relevant in the presence of the complex I substrates, having observed that the LHON cybrid cell line carrying haplogroup U was still the most resistant, whereas no difference between cybrid cell lines was observed with the complex II substrate succinate. One conclusion that we can draw from this first round of experiments is that only the co-presence of the mtDNA pathogenic mutation for LHON with different mtDNA haplogroups leads to the emergence of their modifying role. In other words, the co-occurrence of the primary LHON mutations 11778/ND4 and 14484/ND6 with the haplogroup J1 background further enhances cybrid sensitivity to the neurotoxin 2,5-HD.

To confirm these results on the cybrid cell system, we run the same experiments on fibroblasts obtained from controls and LHON patients. Despite the variable nuclear background, we observed the same specific hypersensitivity to 2,5-HD toxicity with the LHON/haplogroup J1 fibroblasts (11778/ND4 and 14484/ND6 mutations). It is worthy of note that in this case the direct comparison of two LHON cases carrying both the 14484/ND6 mutation, one on haplogroup V and one on haplogroup J1, showed that only the combination 14484/J is hypersensitive to the toxicant. It is of further note that the fibroblasts with the 11778/ND4 mutation on haplogroup H mtDNA showed high resistance to the toxic effect of 2,5-HD. The fact that two unrelated cases with the 11778/ND4 mutation (the cybrid with a haplogroup U mtDNA and the fibroblasts with a haplogroup H mtDNA) were both hyper-resistant to the toxicant prompts the speculation that the 11778/ND4 mutation itself confers this resistance, whereas its combination with haplogroup J1 confers hyper-sensitivity. Further support to this hypothesis derives by the direct comparison of the LHON fibroblasts carrying the 11778/ND4 mutation on a haplogroup H mtDNA with the companion control fibroblasts with a mtDNA belonging to the same haplogroup, the latter failing to show any resistance to 2,5-HD toxicity. On the other hand also the 3460/ND1 mutation on haplogroups H (cybrids) and U (fibroblasts) did not show differences in 2,5-HD sensitivity relative to controls, thus supporting our hypothesis of a direct role for the 11778/ND4 mutation in 2,5-HD resistance.

The functional consequences of the non-synonymous variants clustered on haplogroup J, involving both complex I ND subunit genes and *cytochrome b* (*cyt b*), the only mtDNA-encoded subunit of complex III, remain poorly understood. Haplogroups J and T share a common root characterized by the 4216/ND1 and 15452/*cyt b* ancient polymorphisms [4], [44]. Haplogroups J and T then diverged by acquiring the 13708/ND5+10398/ND3 and the 4917/ND2 polymorphisms, respectively [4], [44]. Therefore, the 13708/ND5 as well as the 10398/ND3 variants are still ancient root mutations, predating the divergence of haplogroup J into subclades. Some of these clades, J1c and J2b in particular, which have accumulated additional and more recent non-synonymous nucleotide changes (14798/*cyt b* for J1c and 15257/*cyt b*+15812/*cy tb* for J2b), have been associated with an increased penetrance of the 11778/ND4 and 14484/ND6 mutations [4], [5], [7]. The specific role played by all these different ND+*cyt b* variants clustered in J1c and J2b subclades of haplogroup J needs to be properly investigated [4], [7]. However, there is also a deeper level of molecular complexity that needs to be considered. This is constituted by other non-synonymous changes, often defined as “private” or “almost private” mutations, which because of their very low population frequencies are not (yet) reported in any of the currently available databases for mtDNA variation, and for which a functional role in some cases is not unlikely. The sequencing of the entire mitochondrial genome allowed us to identify all non-synonymous changes in our cell lines (Table 1). We found out, for instance, that the HL/F29L fibroblasts and the relate cybrid HL180 carry the 7042T>C/COI and the 14279G>A/ND6 mutations together with the 14484/ND6 canonical LHON mutation [7]. Similarly the 9145G>A/ATP6 non-synonymous change is present in the FJ/F08L fibroblasts and the combination of 7904A>G/COII+14249G>A/ND6 changes characterizes the RA/F07L fibroblasts. At the moment, it is not possible to predict the effect, if any, for these non-synonymous variants, especially taking into account that their specific effects could be modulated by the mtDNA background. However, one noticeable feature that is evident from Table 1 is that all cells carrying a haplogroup J mtDNA harbor an extensive accumulation of non-synonymous variants. This finding may explain the special behaviour of haplogroup J in modulating the 2,5-HD toxicity, as shown by the current study, and in influencing LHON penetrance as previously reported [4], [5].

In both cell systems, cybrids and fibroblasts, we also tested the combined effect of two toxicants, adding toluene to 2,5-HD, as we recently documented that humans may suffer exposure to a mixture of solvents [20]. In this case the synergic action of the two toxicants was demonstrated in most cell lines (cybrids and fibroblasts), but doing so we minimized most of the differences due to the mtDNA background. In particular, it must be noticed that all the LHON cell lines on the haplogroup J1c were already hypersensitive to 2,5-HD, and the addition of toluene did not change their viability, whereas all control cells with haplogroup J1c did show a further, significant depression of their viability. By experimenting different conditions, we have noted that the interaction of toluene with 2,5-HD was essentially based on the fact that 2,5-HD increases the solubility of toluene in the medium compared to the addition of toluene alone. The synergic effect of the co-exposure to both toxicants becomes particularly relevant when we consider those cells carrying the 11778/ND4 mutation on haplogroups U or H, which were resistant to 2,5-HD alone, but decreased their viability as those with haplogroup J1c after adding toluene (Figure 4). This applies directly to the LHON patient we previously reported, which carried the 11778/ND4 mutation on a haplogroup H and was exposed at least to both n-hexane and toluene [20].

The results of the present *in vitro* study, which was designed only for a “proof of principle” demonstration, not necessarily can be directly translated into the effects of real-life exposure to 2,5-HD in human subjects genetically predisposed to LHON (or to other mitochondrial diseases). The absence of an animal model for LHON greatly limits more extensive investigations. However, it should be noticed that the concentrations of 2,5-HD used in the present study were much higher than would be encountered in real-world situations, such as those where occupational exposure to n-hexane metabolite (of which the 2,5-HD is the toxic metabolite) can lead to neuropathy [45]. However, it is also unknown how these toxicants may be absorbed, transported and accumulated both in cultured cells(cybrids and fibroblasts) or *in vivo* tissues and in particular, the concentrations achieved in mitochondria of retinal ganglion cells in the retina. Moreover, the limit values proposed for the occupational oxposure to n-hexane are calculated on the basis of is capability to produce, through 2,5-HD, axonal damages but nothing is known about the exposure level can lead to death of fibroblasts or other cells.

The proof of principle provided by the present study, demonstrating that mtDNA haplogroups may interact with environmental factors, has broad implications. For example, exposure to environmental factors, either toxic as tobacco smoke or the complex I inhibitor rotenone widely used as pesticide, or even just variations in diet, have all been variously implicated in predisposition to cancer or late onset neurodegenerative diseases such as Parkinson (PD) and Alzheimer (AD) diseases [46]. In the case of PD there are multiple analogies with LHON. For PD there is converging evidence that defective complex I underlies part of the pathogenic mechanism. In fact, it has been experimentally shown that 1-methyl 4-phenyl 1,2,3,6-tetrahydropyridine (MPTP) and rotenone exposure, both complex I inhibitors, may induce a disorder closely mimicking PD in humans and animals [47]–[50]. Based on this evidence, it has been proposed that the wide use of rotenone as pesticide and its environmental presence may underlie a subset of the sporadic PD cases in the US [50]. Furthermore, certain single nucleotide polymorphisms or a haplogroup have been suggested to play a modifying role [51], [52]. These considerations put Parkinson disease on the spot for being another candidate disease to investigate the possible interaction of environmental exposure to toxics and mtDNA haplogroups in modulating protection or predisposition to develop the disease. Similar scenarios may be envisaged for mtDNA haplogroup/diet interactions in high impact diseases such as hypertension, obesity, diabetes and cancer [53]. Careful evaluations performed by combining large scale epidemiological investigation with *in vitro* cell studies may prove a powerful tool to highlight specific genetic/environmental interactions relevant for the pathogenic or protective mechanisms in these very frequent disorders.

## Materials and Methods

### Materials

3-[4,5-dimethylthiazol-2-yl]-2,5-diphenyl tetrazolium bromide (MTT), oligomycin, rotenone, pyruvate, malate, succinate, ATP monitoring kit, toluene and 2,5HD were purchased from Sigma-Aldrich (Milan, Italy).

### Sequence Variation of mtDNAs and Haplogroup Affiliation

The three LHON mtDNA pathogenic mutations (11778/ND4, 3460/ND1 and 14484/ND6) were screened in LHON cybrids and fibroblasts by standard PCR amplification of convenient mtDNA fragments followed by digestion with appropriate restriction enzymes [44].

The mtDNA sequence variation and haplogroup affiliation of cybrids and fibroblasts, except those already characterized [7], [54], was determined by sequencing their entire mitochondrial genome as previously reported [4].

### Cells and Culture Condition

Transmitochondrial cytoplasmic hybrids (cybrids) were generated as previously reported from control donor and LHON fibroblasts [34]. Cybrids were grown in Dulbecco's modified Eagle's medium (DMEM) supplemented with 10% fetal calf serum (South America source from Gibco, Invitrogen, Italy), 2 mM L-glutamine, 100 U/ml penicillin, 100 µg/ml streptomycin and 0.1 mg/ml bromodeoxyuridine, in an incubator with a humidified atmosphere of 5% CO_2_ at 37°C. Skin fibroblasts were derived, following informed consent, from three healthy donors and six LHON patients from unrelated families bearing the 11778/ND4, 3460/ND1 and 14484/ND6 mutations. Fibroblasts were grown in DMEM supplemented with 10% fetal bovine serum, 2 mM L-glutamine and antibiotics. For the experiments, 4×10^4^ cells were seeded in 24-well dishes and incubated for 24 hours with 2,5-HD alone, or after toluene addition.

### Cell Viability Measurement

The percentage of viable cells was measured with the colorimetric 3-(4,5-dimethyl thiazol-2yl)-2,5-diphenyl tetrazolium bromide (MTT) assay, as previously described [35]. Briefly, after 24 hours incubation with different amount of 2,5-HD, 0.5 mg/ml MTT was added to the medium and after 3 hours, 5% SDS and 5 mM HCl were added to solubilize the formazan salt crystals. Absorbance was measured using a VICTOR3 Multilabel Plate Counter (PerkinElmer Life and Analytical Sciences, Zaventem Belgium) with a 570 nm filter.

### ATP Synthesis Assay and Inhibition by 2,5-HD

The assay of mitochondrial ATP synthesis was performed according to Manfredi et al. [55], with minor modifications. Briefly, after trypsinization, cells (10×10^6^/ml) were resuspended in buffer A (10 mM KCl, 25 mM Tris-HCl, 2 mM EDTA, 0.1% BSA, 10 mM potassium-phosphate, 0.1 mM MgCl_2_, pH 7.4), kept for 15 min at room temperature, and then incubated with 50 µg/ml digitonin for 1 min. After centrifugation, the cell pellet was resuspended in buffer A and aliquots were taken to measure ATP synthesis, protein content [56] and citrate synthase activity [57]. Aliquots of cells (0.1–0.2 mg protein) were incubated with 5 mM malate plus 5 mM glutamate (complex I substrates) or with 10 mM succinate plus 2 µg/ml rotenone (complex II substrate) in the presence or absence of 10 mg/ml 2,5-HD. The reaction was started by addition of 0.2 mM ADP in the presence of luciferine/luciferase, as detailed by the manufacturer's instructions, and chemiluminescence was determined as a function of time with a luminometer. After addition of 10 µM oligomycin, the chemiluminescence signal was calibrated with an internal ATP standard. The residual activity of ATP synthesis after addition of 2,5-HD was expressed as percentage of the activity of untreated cells.

### Statistical Analysis

Data, expressed as means of at least three determinations±SD, were analyzed using One Way ANOVA followed by the Holm-Sidak method. Values for each LHON cybrid cell line were analysed versus grouped values of all control cybrids using the Student's *t* test. P values<0.05 were considered significant.
